# Prevalence and Impact of Protein-Calorie Malnutrition in Hospitalized Patients with Steatotic Liver Disease

**DOI:** 10.21203/rs.3.rs-8080670/v1

**Published:** 2025-12-16

**Authors:** Alexandra C. Greb, Sowon Kim, Andrew Roney, Peng-Sheng Ting, Berkeley N. Limketkai, Po-Hung Chen

**Affiliations:** University of California, Los Angeles; Olive View-UCLA Medical Center; University of California, Los Angeles; Tulane University; University of California, Los Angeles; Johns Hopkins University

**Keywords:** Protein Calorie Malnutrition (PCM), Alcohol-Associated Liver Disease (ALD), Metabolic Dysfunction-Associated Steatotic Liver Disease (MASLD), National (Nationwide) Inpatient Sample (NIS)

## Abstract

**Purpose:**

Patients with liver disease often experience nutritional insufficiency due to an interplay of metabolic disturbances and dietary alterations leading to decreased muscle mass and the development of protein-calorie malnutrition (PCM). This study aimed to evaluate the prevalence of PCM in patients with steatotic liver disease (alcohol associated liver disease (ALD) and metabolic dysfunction-associated steatotic liver disease (MASLD)) and their impacts on mortality and healthcare utilization.

**Methods:**

We identified hospitalizations with ALD, MASLD, and PCM using International Classification of Diseases codes in the National Inpatient Sample from 2016 to 2020. Descriptive analyses compared hospitalizations with and without PCM. Multivariable linear models adjusting for confounders evaluated the association between PCM and inpatient mortality, length of stay (LOS), and total charges.

**Results:**

PCM was found to be significantly more prevalent among hospitalizations with ALD or MASLD than those with neither (ALD: 175.5 vs 51.7; MASLD: 69.2 vs 52.9; neither: 51.5 per 1000 hospitalizations; P < 0.001). Among hospitalizations with ALD or MASLD, PCM was significantly associated with higher mortality (ALD: adjusted odds ratio [aOR] 1.85, 95% CI 1.79–1.91; MASLD: aOR 2.30, 95% CI 2.10–2.52), LOS (ALD: 3.91 days, 95% CI 3.80–4.01; MASLD: 5.17 days, 95% CI 5.00–5.33), and total charges (ALD: $47k higher charges, 95% CI $45k–$50k; MASLD: $60k higher charges, 95% CI $57k–$64k).

**Conclusion:**

We found a higher prevalence of PCM among individuals with ALD compared to those with MASLD or neither condition. PCM was associated with increased mortality, LOS, and total charges in those with ALD and MASLD. Our findings underscore the importance of early identification and management of PCM in patients with steatotic liver disease to mitigate adverse outcomes and reduce healthcare utilization.

## INTRODUCTION

Steatotic liver disease (SLD) is an umbrella diagnosis that includes alcohol-associated liver disease (ALD), metabolic dysfunction-associated steatotic liver disease (MASLD), metabolic dysfunction-associated alcohol-related liver disease (MetALD), and cryptogenic SLD [1]. SLD is characterized by excessive fat accumulation in liver cells and represents the most common form of chronic liver disease.

ALD encompasses a spectrum of liver injury resulting from excessive alcohol intake, while MASLD is diagnosed when an individual with hepatic steatosis meets at least one of five cardiometabolic criteria: overweight, elevated blood glucose, elevated triglycerides, elevated blood pressure, and low high-density lipoprotein [1]. The progression of steatosis to steatohepatitis is an important distinction with increasing inflammatory changes and subsequent progressive liver fibrosis, which can progress to cirrhosis and end-stage liver disease [1,2]. In the United States, end-stage liver fibrosis from ALD and MASLD is rising, whereas the proportion of cirrhosis caused by viral hepatitis (hepatitis B and C) is declining [3]. Understanding the impacts of alcohol- and metabolic dysfunction-related liver diseases is important for addressing the growing burden of advanced liver disease and its complications.

Protein-calorie malnutrition (PCM) is defined as negative changes in body composition and function resulting from malnutrition [4]. PCM is prevalent in patients with cirrhosis and has been shown to affect 27–87% of patients [5,6]. The mechanism of PCM in patients with liver disease is multifactorial, broadly characterized as decreased appetite and nutrient intake, intestinal malabsorption, and increased energy and metabolic expenditure [7–10]. Moreover, PCM in end-stage liver disease is associated with poor outcomes, including increased complications (ascites, esophageal varices, hepatic encephalopathy, hepatorenal syndrome) and lower survival rates as compared to patients without PCM [11–13]. Given the significant risks of comorbid SLD and PCM, our objective was to determine the prevalence of PCM in patients with ALD as compared to patients with MASLD. Additional analyses examined demographic differences between these groups and other clinically relevant outcomes, including in-hospital mortality, length of stay, and total charges.

## METHODS

### Patient Population and Exposures of Interest

In this retrospective study of a nationally representative cohort, hospitalizations with ALD, MASLD, and PCM were identified using the International Classification of Diseases 10th Clinical Modification (ICD-10-CM) codes in the National Inpatient Sample (NIS) from 2016 to 2020. The NIS was developed by the Healthcare Cost and Utilization Project and is the largest publicly available all-payer inpatient database in the United States. The NIS provides a representative sample of hospital discharges, capturing over 90% of all annual inpatient hospitalizations across the country [14]. The Institutional Review Board (IRB) at the University of California, Los Angeles deemed this study exempt from formal review given the nature of the data set: publicly available, de-identified NIS population data. All research was conducted in accordance with both the Declarations of Helsinki and Istanbul.

Inclusion criteria of ALD included ICD-10 code K70.0, K70.1, K70.10, K70.11, K70.2, K703., K70.30, K70.31, K70.4, K70.40, K70.41, and K70.9 but excluded those with MASLD ICD-10 codes of K76.0 or K75.81. The inclusion criteria of MASLD included ICD-10 codes of K76.0 or K75.81 but excluded those with ALD ICD-10 codes of K70 and subgroups. To identify the exposure of interest (PCM), ICD-10 codes of E4 and E64.0 were used to identify patients with PCM (*Supplementary Materials*, Table A).

Demographic and hospitalization-specific information collected for each patient included age, sex, race (White, Black, Hispanic, Asian, Native American, or other), Charlson-Deyo Comorbidity Index (0, 1–2, 3 or greater), elective admission, insurance payer (Medicare, Medicaid, private including HMO, self-pay, no charge, or other), median household income of ZIP code, location of patient’s residence, hospital region (Northeast, Midwest, South, West), bed size of hospital (small, medium, or large), and hospital location (rural, urban teaching, or urban non-teaching). The Charlson-Deyo Comorbidity Index has been validated as a predictor of mortality risk, combining multiple coexisting conditions into a single score to reflect a patient’s overall health burden [15,16].

### Outcomes of Interest

We compared inpatient mortality, length of stay, and total charges between three groups: (1) hospitalized ALD patients with and without PCM, (2) hospitalized MASLD patients with and without PCM, and (3) hospitalized patients with neither ALD nor MASLD with and without PCM. The prevalence of PCM was compared in hospitalized patients with and without ALD or MASLD.

### Statistical Analyses

All analyses incorporated sample weights provided in the dataset to produce nationally representative estimates. Descriptive statistics were used to summarize the demographic and clinical characteristics of each group. Continuous variables were compared using the Student’s t-test, and categorical variables were compared using the chi-square (χ^2^) test.

Multivariable linear and logistic regression models were constructed to evaluate the association between PCM and key inpatient outcomes, including in-hospital mortality, length of stay, and total hospital charges. Models were adjusted for potential confounders, including age, sex, race, medical comorbidities, admission type (elective vs. non-elective), primary payer, median household income by ZIP code, urban-rural residence, hospital region, hospital bed size, and hospital teaching status. All statistical analyses were performed using Stata SE version 17.0 (StataCorp, College Station, TX).

## RESULTS

Among 174,776,205 total hospitalizations, 2,276,264 (1.30%) had ALD and 2,239,145 (1.28%) had MASLD diagnoses. Significant demographic differences, including age, sex, race, Charlson-Deyo index, type of admission, type of insurance, location of patients’ residence and hospital, and hospital size, were observed between PCM and non-PCM groups (Tables 1–3); however, no differences were appreciated in median household income for the ALD and MASLD cohorts (Tables 1 and 2).

**Figure 1:**
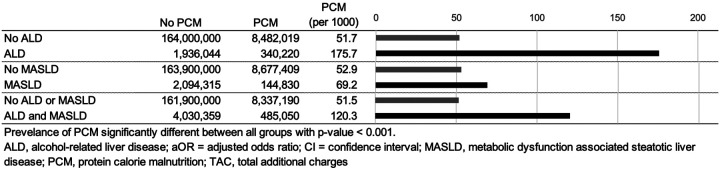
Prevalence of PCM in patients hospitalized with and without ALD, MASLD, or Neither

Among 2,276,264 hospitalizations with ALD, 340,220 (14.9 %) were noted to have PCM, and among 2,239,145 hospitalizations with MASLD, 144,830 (6.47%) were also noted to have PCM. Among 170,237,190 hospitalized patients with neither ALD nor MASLD, 8,337,190 (4.9 %) patients had PCM (Tables 1–3, Figure 1). PCM was significantly more prevalent among hospitalizations with ALD than those without ALD (175.7 vs 51.7 per 1000 hospitalizations; p<0.001). Similarly, PCM was more prevalent among hospitalizations with MASLD than those without MASLD (69.2 vs. 52.9 per 1000 hospitalizations; p<0.001). The prevalence of PCM among hospitalizations with neither ALD nor MASLD was similar to the Neither group, no ALD or MASLD group (Neither: 51.5 vs. No ALD: 51.7 vs. No MASLD: 52.9 per 1000 hospitalizations) (Figure 1).

Multivariable regression models demonstrated that hospitalizations with ALD and PCM experienced increased mortality (adjusted odds ratio [aOR] 1.85; 95% CI: 1.79 – 1.91), length of stay (3.91 more days; 95% CI: 3.80 – 4.01), and total charges ($47,592 additional charges; 95% CI: $45,100 – $50,084) compared to those with ALD but no PCM (Table 4). Compared to patients with MASLD only, patients with MASLD and PCM encountered increased mortality (adjusted odds ratio [aOR] 2.94; 95% CI: 2.76 – 3.12), length of stay (5.17 more days; 95% CI: 5.00 – 5.33), and total charges ($60,552 additional charges; 95% CI: $57,029 – $64,081) (Table 5). Hospitalized patients with neither ALD nor MASLD but PCM had a higher risk of death (adjusted odds ratio [aOR] 2.54; 95% CI: 2.52 – 2.57), increased length of stay (4.91 more days; 95% CI: 4.85 – 4.98), and greater total additional charges ($54,679 additional charges; 95% CI: $52,860 – $56,498) compared to those without ALD, MASLD, or PCM (Table 6).

## DISCUSSION

This retrospective NIS study investigated the prevalence of PCM in patients hospitalized with ALD and MASLD compared to hospitalized patients with neither diagnosis. Other subgroup analyses compared inpatient mortality, length of stay, and total charges between these three groups with and without a diagnosis of PCM. We found that PCM was associated with increased mortality, length of stay, and total charges in those with ALD and MASLD. Further, there was a higher prevalence of PCM among hospitalized patients with ALD or MASLD than those without ALD or MASLD.

When comparing the prevalence of PCM between ALD and MASLD groups in this study, hospitalized patients with ALD were found to have a higher prevalence of PCM than patients with MASLD or neither diagnosis, highlighting both the nutritional and catabolic effects of comorbid alcohol use in developing PCM with end-stage liver disease. Patients with liver disease have a higher risk of developing malnutrition due to a complex interplay of metabolic disturbances, malabsorption, anorexia, and dietary alterations [11]. Previous studies have shown the complex relationship between nutritional status and liver disease, as the liver plays a crucial role in metabolizing most macronutrients, such as protein, carbohydrates, and lipids [6,9]. Adaptations in substrate utilization for energy production can increase the catabolism of protein stores among patients with liver disease, leading to a reliance on skeletal and visceral protein catabolism for energy. This predisposes patients with liver disease to decreased muscle mass and the frequent development of PCM [8,9].

Our multivariable regression models demonstrated that hospitalized patients with SLD and PCM had higher odds ratios of inpatient mortality, longer length of stay, and greater total charges, consistent with previous studies demonstrating an association with mortality and inherent complications (including an increased risk for in-hospital mortality, inpatient charges, and greater length of stay) [17–19]. We also found that PCM is associated with higher in-hospital mortality and resource utilization among hospitalized patients with neither MASLD nor ALD, compared to their counterparts without PCM, supporting the known substantial clinical impact of PCM itself. These findings indicate that PCM independently contributes to worse outcomes in hospitalized patients regardless of coexisting liver disease, consistent with previous studies showing that up to 65% of hospitalized patients experience a poorer nutritional status as compared to healthy individuals [20]. Other research has demonstrated a decline in nutritional status and weight loss as risk factors impacting complications and increased mortality, length of hospital stay, and costs independently of demographic features and disease severity [21,22]. Factors contributing to PCM in this population include illness-related loss of appetite, the fasting period for diagnostic procedures, and acute illness compromising the regular functioning of the digestive system [22,23].

Nutritional disturbances, impaired nutritional absorption, and elevated metabolic needs associated with ALD may explain our finding of a higher prevalence of PCM among patients with ALD compared to MASLD [24,25]. Though both MASLD and ALD share a multifactorial pathogenesis, mainly driven by lipotoxicity from excessive accumulation of lipids and insulin resistance leading to systemic proinflammatory and oxidative stress [26–28], patients with ALD are particularly susceptible to PCM due to the combined physiological impacts of alcohol use and chronic liver inflammation [29]. Reactive oxygen species that are generated during alcohol metabolism may lead to immune response activation and lipid peroxidation, advancing ALD to end-stage liver fibrosis [29,30]. Despite increased caloric intake with alcohol use, patients with ALD are less likely to be obese, hypothesized to be due to an increased resting energy expenditure state [24,31]. Moreover, alcohol has toxic effects on the gastrointestinal tract, leading to impaired digestion and absorption of nutrients [25,32].

Patients with MASLD possess cardiometabolic risk factors such as obesity, insulin resistance, hypertension, and hyperlipidemia. Expectedly, numerous studies have shown that the increasing prevalence of MASLD parallels the increasing prevalence of obesity and obesity-related diseases [33–35]. Hence, it is critical to avoid overnutrition among these patient populations, with clinical guidelines recommending intentional weight loss to help prevent MASLD progression [36,37]. The paradox of concurrent PCM, “the malnourished state,” with obesity, “the over-nourished state,” might not be intuitive. However, patients with MASLD experience chronic inflammation and are likely at risk for malnutrition from increased inflammatory cytokines and oxidative stress, leading to increased adiposity, decreased lean body mass, lipogenesis, and visceral protein catabolism [10,38,39].

Strengths of this study include a large sample size and a nationally representative dataset. Given the large scope of the NIS, we were able to control for relevant demographic and hospitalization-specific factors affecting our outcomes (prevalence of PCM, inpatient mortality, length of stay, and total charges), allowing for more sensitive subgroup analyses. Unfortunately, the NIS does not collect data on descriptive alcohol use, which did not allow for the assessment of alcohol use disorder (AUD) severity in this study. Another consideration of this study is that we did not include patients with MetALD diagnoses. MetALD is a newer term that describes patients with hepatic steatosis who have at least one cardiometabolic risk factor and consume alcohol amounts between 140 and 350 grams per week for females and between 210 and 420 grams per week for males [1]. Due to unsolidified ICD codes for the new nomenclature, we did not investigate the prevalence and clinical impact of PCM among patients with MetALD.

Our nationwide inpatient study demonstrated an increased prevalence of PCM among those with ALD or MASLD compared to hospitalized patients with neither condition. Patients hospitalized with ALD were found to have a higher prevalence of PCM than patients with MASLD, which suggests that additional risks of comorbid alcohol use may contribute to PCM. PCM as an independent risk factor was associated with adverse clinical outcomes in those with ALD, MASLD, or neither. This study emphasizes the importance of regular nutritional assessments in hospitalized patients, conducted by a multidisciplinary team (including a registered dietitian), to implement prompt nutritional interventions and prevent downstream harms associated with PCM. Additionally, clinicians must maintain a low threshold for evaluating patients with MASLD or ALD for PCM, given the multifactorial risk factors associated with developing PCM. Our study highlights the need for future studies to examine PCM in patients with MetALD as well as targeted nutritional interventions that can mitigate the risks associated with malnutrition.

## Supplementary Material

This is a list of supplementary files associated with this preprint. Click to download.

- SUPPLEMENTALTABLES.docx

## Figures and Tables

**Table 1.** Demographic characteristics between hospitalized ALD patients with and without PCM

	No PCM (n =1,936,044)		PCM (n = 340,220)		
					p-value
Age, mean (SD)	53.6	(12.35)	55.7	(11.91)	< 0.001
Female, n (%)	571,555	(0.30)	120,025	(0.35)	< 0.001
Race, n (%)					< 0.001
White	1,267,745	(0.65)	230,060	(0.68)	
Black	186,745	(0.10)	38,385	(0.11)	
Hispanic	306,295	(0.16)	41,750	(0.12)	
Asian	22,680	(0.01)	4,375	(0.01)	
Native American	46,405	(0.02)	6,320	(0.02)	
Other	54,210	(0.03)	8,375	(0.02)	
Charlson-Deyo index, n (%)					< 0.001
0	0	(0.00)	0	(0.00)	
1 to 2	524,445	(0.27)	59,325	(0.17)	
3 or more	1,411,600	(0.73)	280,895	(0.83)	
Elective admissions, n (%)	100,820	(0.05)	14,425	(0.04)	< 0.001
Payer, n (%)					< 0.001
Medicare	561,550	(0.29)	114,140	(0.34)	
Medicaid	671,840	(0.35)	114,910	(0.34)	
Private including HMO	433,165	(0.22)	73,925	(0.22)	
Self-pay	180,670	(0.09)	24,385	(0.07)	
No charge	16,495	(0.01)	2,035	(0.01)	
Other	68,890	(0.04)	10,380	(0.03)	
Median household income of ZIP Code, n (%)					0.146
$1 – $49,999	591,930	(0.31)	105,645	(0.31)	
$50,000 – $64,999	494,785	(0.26)	87,255	(0.26)	
$65,000 – 85,999	447,355	(0.23)	78,950	(0.23)	
$86,000 or more	342,480	(0.18)	58,910	(0.17)	
Location of patient’s residence, n (%)					< 0.001
Central metro	595,195	(0.31)	108,495	(0.32)	
Fringe metro	447,575	(0.23)	73,785	(0.22)	
Pop 250K–1M	419,505	(0.22)	71,690	(0.21)	
Pop 50K–250K	180,775	(0.09)	32,740	(0.10)	
Micropolitan	158,755	(0.08)	29,655	(0.09)	
Not metro or micro	104,370	(0.05)	19,320	(0.06)	
Hospital region, n (%)					< 0.001
Northeast	358,530	(0.19)	50,875	(0.15)	
Midwest	408,590	(0.21)	86,555	(0.25)	
South	674,935	(0.35)	111,965	(0.33)	
West	493,989	(0.26)	90,825	(0.27)	
Bed size of hospital, n (%)					< 0.001
Small	396,964	(0.21)	63,925	(0.19)	
Medium	569,614	(0.29)	94,245	(0.28)	
Large	969,465	(0.50)	182,050	(0.54)	
Hospital location, n (%)					< 0.001
Rural	136,445	(0.07)	20,945	(0.06)	
Urban non-teaching	419,810	(0.22)	67,190	(0.20)	
Urban teaching	1,379,790	(0.71)	252,085	(0.74)	

ALD, alcohol-related liver disease; PCM, protein-calorie malnutrition; SD, standard deviation

**Table 2.** Demographic characteristics between hospitalized MASLD patients with and without PCM

	No PCM (n = 2,094,315)		PCM (n = 144,830)		
					p-value
Age, mean (SD)	55.7	(15.92)	60.0	(15.34)	< 0.001
Female, n (%)	1,154,960	(0.55)	82,480	(0.57)	< 0.001
Race, n (%)					< 0.001
White	1,389,260	(0.66)	98,405	(0.68)	
Black	191,725	(0.09)	15,635	(0.11)	
Hispanic	332,855	(0.16)	17,735	(0.12)	
Asian	47,570	(0.02)	3,580	(0.02)	
Native American	18,335	(0.01)	1,250	(0.01)	
Other	64,100	(0.03)	4,095	(0.03)	
Charlson-Deyo index, n (%)					< 0.001
0	36,110	(0.02)	1,805	(0.01)	
1 to 2	592,400	(0.28)	25,685	(0.18)	
3 or more	1,465,805	(0.70)	117,340	(0.81)	
Elective admissions, n (%)	342,120	(0.16)	10,400	(0.07)	< 0.001
Payer, n (%)					< 0.001
Medicare	828,150	(0.40)	73,435	(0.51)	
Medicaid	385,080	(0.18)	26,165	(0.18)	
Private including HMO	689,245	(0.33)	35,490	(0.25)	
Self-pay	118,530	(0.06)	5,600	(0.04)	
No charge	11,380	(0.01)	490	(0.00)	
Other	59,215	(0.03)	3,450	(0.02)	
Median household income of ZIP Code, n (%)					0.259
$1 – $49,999	608,145	(0.29)	42,280	(0.29)	
$50,000 – $64,999	556,670	(0.27)	39,020	(0.27)	
$65,000 – 85,999	503,310	(0.24)	34,325	(0.24)	
$86,000 or more	389,210	(0.19)	26,250	(0.18)	
Location of patient’s residence, n (%)					< 0.001
Central metro	614,640	(0.29)	45,270	(0.31)	
Fringe metro	506,730	(0.24)	32,950	(0.23)	
Pop 250K–1M	460,260	(0.22)	29,945	(0.21)	
Pop 50K–250K	198,545	(0.09)	13,980	(0.10)	
Micropolitan	176,295	(0.08)	13,325	(0.09)	
Not metro or micro	127,720	(0.06)	8,475	(0.06)	
Hospital region, n (%)					< 0.001
Northeast	351,580	(0.17)	19,325	(0.13)	
Midwest	451,405	(0.22)	38,835	(0.27)	
South	831,830	(0.40)	52,580	(0.36)	
West	459,499	(0.22)	34,090	(0.24)	
Bed size of hospital, n (%)					< 0.001
Small	418,900	(0.20)	24,495	(0.17)	
Medium	593,590	(0.28)	37,360	(0.26)	
Large	1,081,826	(0.52)	82,975	(0.57)	
Hospital location, n (%)					< 0.001
Rural	141,535	(0.07)	8,170	(0.06)	
Urban non-teaching	430,005	(0.21)	24,405	(0.17)	
Urban teaching	1,522,775	(0.73)	112,255	(0.78)	

MASLD, metabolic dysfunction associated steatotic liver disease; PCM, protein-calorie malnutrition; SD, standard deviation

**Table 3.** Demographic characteristics between hospitalized patients with no ALD or MASLD, with and without PCM

	No PCM (n - 161,900,000)		PCM (n - 8,337,190)		
					p-value
Age, mean (SD)	48.7	(27.68)	66.4	(18.88)	< 0.001
Female, n (%)	91,754,697	(0.57)	4,271,855	(0.51)	< 0.001
Race, n (%)					
White	100,200,000	(0.62)	5,486,760	(0.66)	
Black	24,037,240	(0.15)	1,353,040	(0.16)	
Hispanic	19,758,578	(0.12)	712,000	(0.09)	
Asian	4,891,727	(0.03)	257,745	(0.03)	
Native American	1,023,935	(0.01)	47,695	(0.01)	
Other	5,570,037	(0.03)	229,950	(0.03)	
Charlson-Deyo index, n (%)					< 0.001
0	74,998,750	(0.46)	1,144,415	(0.14)	
1 to 2	27,462,996	(0.17)	1,390,905	(0.17)	
3 or more	59,461,861	(0.37)	5,801,870	(0.70)	
Elective admissions, n (%)	33,965,838	(0.21)	774,180	(0.09)	< 0.001
Payer, n (%)					< 0.001
Medicare	63,539,474	(0.39)	5,427,330	(0.65)	
Medicaid	37,612,631	(0.23)	1,157,930	(0.14)	
Private including HMO	48,637,024	(0.30)	1,353,015	(0.16)	
Self-pay	6,631,380	(0.04)	192,115	(0.02)	
No charge	493,380	(0.00)	15,985	(0.00)	
Other	4,775,453	(0.03)	181,905	(0.02)	
Median household income of ZIP Code, n (%)					< 0.001
$1 – $49,999	48,054,165	(0.30)	2,563,630	(0.31)	
$50,000 – $64,999	41,764,983	(0.26)	2,117,590	(0.25)	
$65,000 – 85,999	37,823,121	(0.23)	1,906,735	(0.23)	
$86,000 or more	31,629,914	(0.20)	1,592,210	(0.19)	
Location of patient’s residence, n (%)					< 0.001
Central metro	47,924,993	(0.30)	2,700,664	(0.32)	
Fringe metro	38,920,718	(0.24)	1,909,615	(0.23)	
Pop 250K–1M	33,647,620	(0.21)	1,649,654	(0.20)	
Pop 50K–250K	14,892,296	(0.09)	759,030	(0.09)	
Micropolitan	14,715,411	(0.09)	749,665	(0.09)	
Not metro or micro	10,984,733	(0.07)	515,775	(0.06)	
Hospital region, n (%)					< 0.001
Northeast	29,606,599	(0.18)	1,448,290	(0.17)	
Midwest	35,732,468	(0.22)	2,022,421	(0.24)	
South	64,457,150	(0.40)	3,067,286	(0.37)	
West	32,127,390	(0.20)	1,799,193	(0.22)	
Bed size of hospital, n (%)					< 0.001
Small	33,799,404	(0.21)	1,579,109	(0.19)	
Medium	46,918,945	(0.29)	2,300,759	(0.28)	
Large	81,205,258	(0.50)	4,457,322	(0.53)	
Hospital location, n (%)					
Rural	14,408,195	(0.09)	592,486	(0.07)	< 0.001
Urban non-teaching	33,386,774	(0.21)	1,655,681	(0.20)	
Urban teaching	114,100,000	(0.70)	6,089,023	(0.73)	

ALD, alcohol-related liver disease; MASLD, metabolic dysfunction associated steatotic liver disease; PCM = protein calorie malnutrition; SD, standard deviation

**Table 4.** Multivariable regression of patients hospitalized with ALD comparing PCM to no PCM

		95% CI			P-Value
Death (aOR)	1.85	1.79	-	1.91	< 0.001
Length of stay (days)	3.91	3.80	-	4.01	< 0.001
Total additional charges (dollars)	$47,592.46	$45,100.51	-	$50,084.41	< 0.001

aOR = adjusted odds ratio; ALD, alcohol-related liver disease; CI = confidence interval; PCM = protein calorie malnutrition

**Table 5.** Multivariable regression of patients hospitalized with MASLD comparing PCM to no PCM

		95% CI			P-Value
Death (aOR)	2.94	2.76	-	3.12	< 0.001
Length of stay (days)	5.17	5.00	-	5.33	< 0.001
Total additional charges (dollars)	$60,555.22	$57,028.99	-	$64,081.44	< 0.001

aOR = adjusted odds ratio; CI = confidence interval; MASLD, metabolic dysfunction associated steatotic liver disease; PCM = protein calorie malnutrition

**Table 6.** Multivariable regression of patients hospitalized without ALD nor MASLD comparing PCM to no PCM

		95% CI			P-Value
Death (aOR)	2.54	2.52	-	2.57	< 0.001
Length of stay (days)	4.91	4.85	-	4.98	< 0.001
Total additional charges (dollars)	$54,679.00	$52,859.67	-	$56,498.33	< 0.001

aOR = adjusted odds ratio; ALD, alcohol-related liver disease; CI = confidence interval; MASLD, metabolic dysfunction associated steatotic liver disease; PCM = protein calorie malnutrition

## Data Availability

Data is provided within the manuscript or supplementary information files.
